# Structure-Thermodynamics-Antioxidant Activity Relationships of Selected Natural Phenolic Acids and Derivatives: An Experimental and Theoretical Evaluation

**DOI:** 10.1371/journal.pone.0121276

**Published:** 2015-03-24

**Authors:** Yuzhen Chen, Huizhi Xiao, Jie Zheng, Guizhao Liang

**Affiliations:** 1 School of Mathematical Sciences, Henan Institute of Science and Technology, Xinxiang 453003, P. R. China; 2 Key Laboratory of Biorheological Science and Technology, Ministry of Education, School of Bioengineering, Chongqing University, Chongqing 400044, P. R. China; 3 Department of Chemical and Biomolecular Engineering, The University of Akron, Akron, Ohio 44325, United States of America; University of Calgary, CANADA

## Abstract

Phenolic acids and derivatives have potential biological functions, however, little is known about the structure-activity relationships and the underlying action mechanisms of these phenolic acids to date. Herein we investigate the structure-thermodynamics-antioxidant relationships of 20 natural phenolic acids and derivatives using DPPH^•^ scavenging assay, density functional theory calculations at the B3LYP/6-311++G(d,p) levels of theory, and quantitative structure-activity relationship (QSAR) modeling. Three main working mechanisms (HAT, SETPT and SPLET) are explored in four micro-environments (gas-phase, benzene, water and ethanol). Computed thermodynamics parameters (BDE, IP, PDE, PA and ETE) are compared with the experimental radical scavenging activities against DPPH^•^. Available theoretical and experimental investigations have demonstrated that the extended delocalization and intra-molecular hydrogen bonds are the two main contributions to the stability of the radicals. The C = O or C = C in COOH, COOR, C = CCOOH and C = CCOOR groups, and orthodiphenolic functionalities are shown to favorably stabilize the specific radical species to enhance the radical scavenging activities, while the presence of the single OH in the *ortho* position of the COOH group disfavors the activities. HAT is the thermodynamically preferred mechanism in the gas phase and benzene, whereas SPLET in water and ethanol. Furthermore, our QSAR models robustly represent the structure-activity relationships of these explored compounds in polar media.

## Introduction

Phenolic acids and derivatives are a subclass of a larger category of metabolites (commonly named as “phenolics”), which widely spread throughout the plant kingdom [1]. Although the basic skeleton containing a carboxylic acid function and a phenolic ring remains the same [1,2], the numbers and positions of hydroxyl groups and other substituents on the aromatic ring create significant variations in both structures and functions. Recent interests in phenolic acids and derivatives mainly come from their potential biological functions, such as anti-inflammatory, antiallergic, antimicrobial, anticarcinogenic and antiviral activities [2]. However, little is known about the detailed action mechanisms of this class of compounds [1,3]. Thus, a thorough investigation of approximately 8000 naturally occurring phenolics [2] will help us to identify the compounds with desirable functionalities important for public health [4].

Phenolic compounds generally exert their protective activities by three different mechanisms [5–9]: hydrogen atom transfer (HAT) (Eq 1), electron transfer-proton transfer (SETPT) (Eq 2 and 3), and sequential proton loss-electron transfer (SPLET) (Eq 4, 5 and 6). All the mechanisms are believed to play important roles in determining radical scavenging activities of antioxidants in various environmental conditions [5,10]. It has been shown that radical scavenging activities of phenolic antioxidants are related to the phenolic O-H bond dissociation enthalpy (BDE), ionization potential (IP), proton dissociation enthalpy (PDE), proton affinity (PA) and electron transfer enthalpy (ETE) [6,10–14].

$$ArOH+R^{•}\to ArO^{•}+RH$$ (1)

$$ArOH+R^{•}\to ArOH^{•+}+R^{-}$$ (2)

$$ArOH^{•+}+R^{-}\to ArO^{•}+RH$$ (3)

$$ArOH\to ArO^{-}+H^{+}$$ (4)

$$ArO^{-}+R^{•}\to ArO^{•}+R^{-}$$ (5)

$$R^{-}+H^{+}\to RH$$ (6)

Radical scavenging activities of phenolic antioxidants are largely influenced by their structural and environmental features in vivo [5]. In vitro determination of radical scavenging activities has often relied on the ability of phenolic compounds to quench the color of stable radicals such as the 2,2-diphenyl-1-picrylhydrazyl (DPPH) radical [15,16]. This reaction with DPPH is considerably affected by experimental conditions including the absolute and relative concentrations of DPPH and antioxidants, solvents, hydrogen bonding strength, room temperature, time, and unspecified pH [16]. Accordingly, the “one-size-fits-all” radical scavenging mechanisms may or may not work, or a combination of several mechanisms may provide a better explanation for radical scavenging activities of phenolic antioxidants [16]. Besides, lack of standardization in sample preparation, reaction conditions, analytical protocols and expression of antioxidant action leads to difficulty or impossibility to compare different results obtained from different laboratories.

The quantum chemistry and computation methodologies allow obtaining atomic-level structures and energetic information of the systems with accuracy equivalent to or greater than those obtained from experiments. Therefore, theoretical calculations have been widely used as a cogent tool for rational design of novel potential drugs and for investigation of the underlying structure-activity relationships of these drugs [12]. There are several successful examples of rational interpretation of structure-activity relationships of some natural antioxidants [11–13,17,18] and design of novel antioxidants [6,19,20] using powerful and economical quantum chemical methods especially density functional theory (DFT). It has been reported that since most of the DFT methods underestimate thermodynamics parameters such as BDEs, these methods are more reliable and suitable for relative calculations than for absolute calculations [21].

In parallel, quantitative structure-activity relationship (QSAR) is one of powerful computational methods for prediction of activities, determination of action mechanisms, design of drugs, materials, catalysts, and proteins/peptides with desirable activities and functions [22–28]. The QSAR methods have been used to explore the structure-activity relationships and action mechanisms of phenolic compounds [29]. A predictive and interpretable QSAR model can help to further understand mechanisms of action of the explored molecules toward the target systems. Since chemical descriptors are the core of QSAR modeling, significant efforts and progress have been made to develop a wide variety of chemical descriptors to describe different levels of chemical, physical, and structural characteristics of the target molecules/systems [27]. The comprehensibly physiochemical descriptors, i.e., BDE, IP, PDE, PA and ETE, calculated by DFT calculations have been employed to elucidate the structure-activity relationships of the investigated phenolic antioxidants [6,10,13,30]. Correlation methods are the second important part of QSAR modeling. A linear model has some advantages such as simplicity and interpretability over a non-interpretably nonlinear one [27], ensuring the interpretability of QSAR models.

In this work, we investigated the structure-thermodynamics-antioxidant activity relationships of 20 natural phenolic acids and derivatives using combined experimental and computational approaches. The antioxidant activities of the compounds were evaluated for their total antioxidant capacity using the DPPH^•^ scavenging assay. With high-precision DFT calculations, five sets of thermodynamics parameters (BDE, IP, PDE, PA and ETE) were identified and used to evaluate three working mechanisms (HAT, SETPT and SPLET) under different micro-environments (gas-phase, benzene, water and ethanol). Moreover, the spin density in free radicals, as well as the highest occupied molecular orbital (HOMO) distribution, was also computed to better describe the radical scavenging reactivity of the studied molecules. We also established the QSAR models to characterize the structure-activity relationships of the antioxidants. This work provides structural-based insights into the action mechanisms of phenolics, which may help expand their applications in pharmaceutical and food science.

## Principles and Methods

### Chemicals

DPPH was purchased from Sigma Chemical Co. A total of 20 naturally occurring phenolic acids and derivatives (Table A in S1 File) were purchased from Aladdin Industrial Inc. These compounds contain 16 natural phenolic acids, with two distinguishing constitutive carbon frameworks: COOH and C = CCOOH, and 4 compounds referred to as phenolic acid derivatives, with a COOR structure, including propyl gallate (01), methyl gallate (09), methyl vanillate (15), and ferulic acid ethyl ester (18).

### DPPH radical scavenging assay

The DPPH assay has been widely used for the measurement of free radical scavenging capacity of various natural products [15,16]. The DPPH radical is a stable organic free radical with adsorption band at 515–528 nm. It loses this adsorption when accepting an electron or a free radical species, which results in a visually noticeable discoloration from purple to yellow [31]. The DPPH radical scavenging assay was measured using the method of Sun and Ho [15]. Briefly, 2 mL DPPH solution (0.2 mmol/L, in ethanol) was incubated with different concentrations of the sample. The reaction mixture was shaken and incubated in the dark for 30 min at room temperature. The absorbance was read at 517 nm against ethanol. The control containing ethanol instead of the sample and the blank containing ethanol instead of DPPH solution were also made. The test was run in triplicate and the inhibition of the DPPH radical of the sample was calculated according to the following formula:

$$\text{DPPH scavenging activity}\;\left(\%\right)=\frac{{\text{Abs.}}_{\text{control}}-\left({\text{Abs.}}_{\text{sample}}-{\text{Abs.}}_{\text{blank}}\right)}{{\text{Abs.}}_{\text{control}}}\times 100\%$$

The percentage of DPPH radical scavenging activity was plotted against the sample concentration to acquire the IC_50_ value, defined as the concentration of sample necessary to cause 50% inhibition. The DPPH measurements were assayed using a spectrophotometer (UNICO7200, Unico(Shanghai) Instrument Co., Ltd.).

### DFT calculations

All geometries were completely optimized in all internal degrees of freedom using DFT calculations with the B3LYP [32] functional and 6-311++G(d,p) [33] basis set. For all of the radical systems, the unrestricted B3LYP/6-311++G(d,p) method was used. Vibrational frequencies were further computed at the same level to ensure no imaginary frequency for the optimized structures. For open-shell species, accuracy of the energy evaluation is sensitive to spin contamination. Here, spin contaminations of radicals were found in the 0.76–0.78 range, and then spin contaminants dropped to a correct value of 0.75 after the annihilation of the first spin contaminant.

Here, BDE, IP, PDE, PA and ETE were determined in the gas phase, benzene, water and ethanol solvents at 298 K based on the following expressions (Eq 7, 8, 9, 10 and 11):

$$BDE=H(ArO^{•})+H(H^{•})-H(ArOH)$$ (7)

$$IP=H\left(ArOH^{•+}\right)+H\left(e^{-}\right)-H\left(ArOH\right)$$ (8)

$$PDE=H\left(ArO^{•}\right)+H\left(H^{+}\right)-H\left(ArOH^{•+}\right)$$ (9)

$$PA=H\left(ArO^{-}\right)+H\left(H^{+}\right)-H\left(ArOH\right)$$ (10)

$$ETE=H\left(ArO^{•}\right)+H\left(e^{-}\right)-H\left(ArO^{-}\right)$$ (11)

Therein, the BDE values were used to estimate the reactivity of an ArOH in HAT. The IP and PDE values from the ArOH^•+^ radical cation were calculated to describe the SETPT mechanism. The PA values of the phenoxide anion, ArO^−^, were used to characterize the reaction enthalpy of the first step, and ETEs for the reaction enthalpy of the following step, in the SPLET mechanism.

The solvent effects were computed using an integral equation formalism polarized continuum model IEF-PCM method [34]. The calculated gas-phase enthalpy of a proton and an electron is 1.483 and 0.752 kcal/mol, respectively [35]. Proton and electron solvation enthalpies were taken from Rimarcik et al.’s report [36], and hydrogen atom solvation enthalpies from Parker’s [37] and Bizarro et al’s report [38]. All calculations were performed using Gaussian 09 [39].

### QSAR modeling and validation

Simple or multiple linear regression analysis (SLR or MLR) was performed to derive the QSAR models. The BDE, IP, PDE, PA and ETE descriptors were regarded as inputs of the models, and the negative logarithmic IC_50_ values (pIC_50_) were treated as dependent variables. The BDE-antioxidant activity relationships were described by SLR-based QSAR models, while the IP-PDE-antioxidant activity and the PA-ETE-antioxidant activity relationships were described by MLR-based models. The leave-one-out [40] cross validation was employed to test the predictive ability of the models. The modeling performance was assessed based on a serial of statistical parameters, including multiple correlation coefficients (*R* ^2^ for regression modeling and *Q* ^2^ for leave-one-out cross validation), standard error, and Fisher’s criterion, etc. The linear regression operation and the leave-one-out cross validation was performed by our in-house applied program.

## Results and Discussion

### Effects of intra-molecular hydrogen bonds on the stability of the radicals

**Fig 1:**
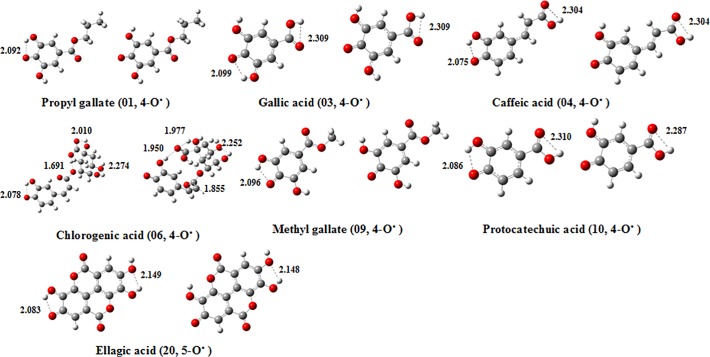
Optimized structures of 7 compounds with orthodiphenolic functionalities calculated at the B3LYP/6-311++G(d,p) level in ethanol. (left: a hydrogen bond is formed between the 4-O^•^ center and the *meta* OH. right: no hydrogen bond is formed between the 4-O^•^ center and the *meta* OH.)

**Fig 2:**
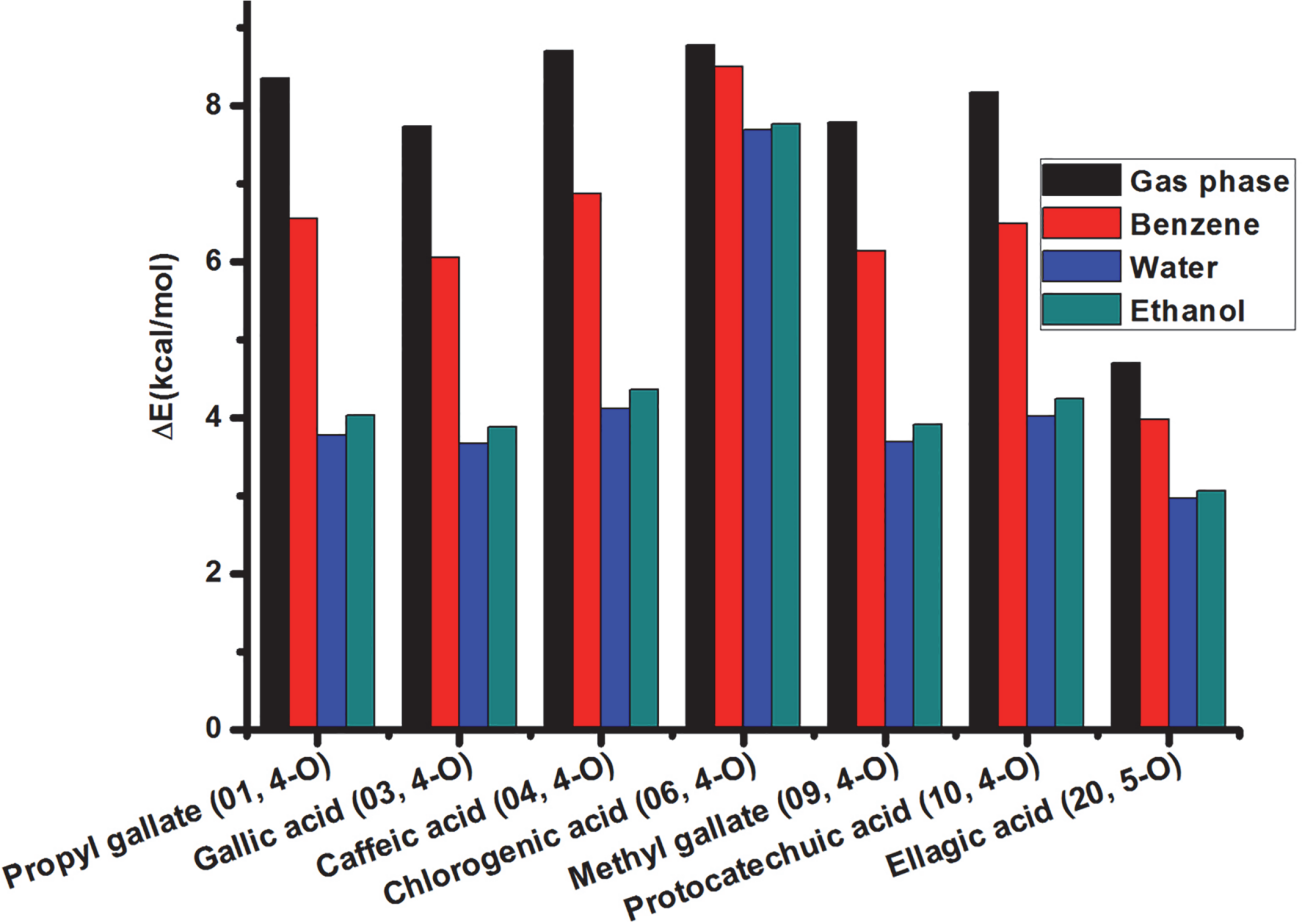
The energy difference (ΔE in kcal/mol) caused by the hydrogen bond between the O^•^ center and the *meta* OH for 7 radicals calculated at the B3LYP/6-311++G(d,p) levels of theory in 4 reaction media.

Considering that hydrogen bonds play an important role in the stability of the studied parent phenolic compounds and their corresponding hydrogen atom-abstracted radicals, we comparably calculated hydrogen bonds between the phenol-O^•^ and the *meta* OH of both the radicals and their parent molecules. The energies of the optimized 7 parent molecules (Fig. A in S1 File) with hydrogen bond(s) between the phenol-OH and the *meta* OH are reduced by 3.1–8.7 kcal/mol relative to those of 7 parent molecules without hydrogen bond(s) between the phenol-OH and the *meta* OH. The corresponding radicals derived from 7 parent compounds display that a 4-O^•^···H-O-3 hydrogen bond is formed between the 4-O^•^ center and its neighboring OH group (Fig. 1), demonstrating an important contribution to the stability of the radicals. The energy difference (Fig. 2) of the radicals optimized at the B3LYP/6-311++G(d,p) level shows that among 4 different reaction conditions, the relative energies of the 4-O^•^ centers forming a hydrogen bond with the *meta* OH are less than those of the 4-O^•^ centers without any hydrogen bond. This supported hydrogen bond(s) could help to stabilize these radicals and be beneficial to the radical scavenging capacity of the parent molecules. Thus, in comparison to vanillic acid (02), an O-H···O hydrogen bond between the O^•^ center and the *meta* OH in protocatechuic acid (10) was formed, thereby to stabilize the corresponding phenoxy radical, which could partly explain why the former has a low radical scavenging activity (pIC_50_ = 2.44) relative to the latter (pIC_50_ = 4.25). More importantly, the largest energy differences between the radicals with hydrogen bonds and without hydrogen bonds derived from one same compound were found in the gas phase, whereas the smallest ones in water (Fig. 2). We speculated that water molecules are likely to form strong hydrogen bonds with the corresponding 4-O^•^, thereby to conductively stabilize the corresponding radicals.

### Structure-BDE-activity relationships

We calculated a total of 31 O-H BDE values generated from 20 parent phenolic acids and derivatives with intra-molecular hydrogen bonds at the B3LYP/6-311++G(d,p) level of theory. As displayed in Table 1, the O-H BDE values exhibit an analogous size for each compound whatever the reaction medium. This indicated that from the thermodynamic point of view, the HAT mechanism has a close occurrence probability in 4 reaction media.

**Table 1:** BDEs of 20 investigated phenolic compounds and phenol calculated at the B3LYP/6-311++G(d,p) level ^a^.

No.	Compound	BDE (kcal/mol)				pIC_50_
		Gas	Benzene	Ethanol	Water	
1	Propyl gallate					4.81
	3-OH	85.0	83.8	82.2	82.0	
	4-OH	**77.4**	**77.0**	**76.3**	**76.2**	
	5-OH	78.2	78.5	79.0	79.0	
2	Vanillic acid					2.44
	4-OH	**85.2**	**84.2**	**82.7**	**82.6**	
3	Gallic acid					4.70
	3-OH	77.9	78.5	79.2	79.3	
	4-OH	**78.0**	**77.7**	**77.0**	**76.9**	
	5-OH	85.9	84.6	82.8	82.7	
4	Caffeic acid					4.52
	3-OH	85.4	84.1	82.2	82.0	
	4-OH	**73.7**	**74.3**	**75.1**	**75.2**	
5	Sinapic acid					4.19
	4-OH	**78.1**	**76.8**	**75.0**	**74.8**	
6	Chlorogenic acid					4.37
	3-OH	94.4	93.7	82.4	84.7	
	4-OH	**83.1**	**83.0**	**75.0**	**75.5**	
7	Salicylic acid					1.10
	2-OH	**92.4**	**108.5**	**89.7**	**89.3**	
8	Syringic acid					4.32
	4-OH	**80.8**	**79.6**	**78.0**	**77.8**	
9	Methyl gallate					4.74
	3-OH	85.5	109.9	82.5	82.3	
	4-OH	**77.6**	**77.2**	**76.6**	**76.5**	
	5-OH	77.4	78.0	78.9	78.9	
10	Protocatechuic acid					4.25
	3-OH	85.5	84.3	82.7	82.5	
	4-OH	**76.8**	**77.6**	**78.6**	**78.7**	
11	2,5-Dihydroxybenzoic acid					4.53
	2-OH	**80.0**	**79.5**	**78.7**	**78.6**	
	5-OH	106.7	80.7	79.7	79.6	
12	Ferulic acid					3.57
	4-OH	**82.1**	**81.1**	**79.4**	**79.2**	
13	*p*-Coumaric acid					4.54
	4-OH	**82.0**	**81.9**	**81.7**	**81.7**	
14	3-Methylsalicylic acid					1.15
	2-OH	**90.3**	**89.1**	**87.6**	**87.5**	
15	Methyl Vanillate					4.60
	4-OH	**84.7**	**83.5**	**82.1**	**82.0**	
16	3,5-Dinitro salicylic acid					0.61
	2-OH	**97.6**	**97.5**	**97.2**	**97.1**	
17	Isovanillic acid					2.33
	3-OH	**78.7**	**79.0**	**79.5**	**79.5**	
18	Ferulic Acid Ethyl Ester					3.61
	4-OH	**81.6**	**80.5**	**78.9**	**78.7**	
19	4-Methylsalicylic acid					0.75
	2-OH	**85.0**	**84.8**	**84.5**	**84.5**	
20	Ellagic acid					5.00
	4-OH	84.8	83.9	82.6	82.4	
	5-OH	**77.1**	**77.7**	**78.3**	**78.3**	
21	Phenol	83.6	83.2	82.9	82.9	

^a^ The parameters are calculated based on the optimized structures with intra-molecular hydrogen bonds. The bold parameters are used as independent variables in the QSAR model.

**Fig 3:**
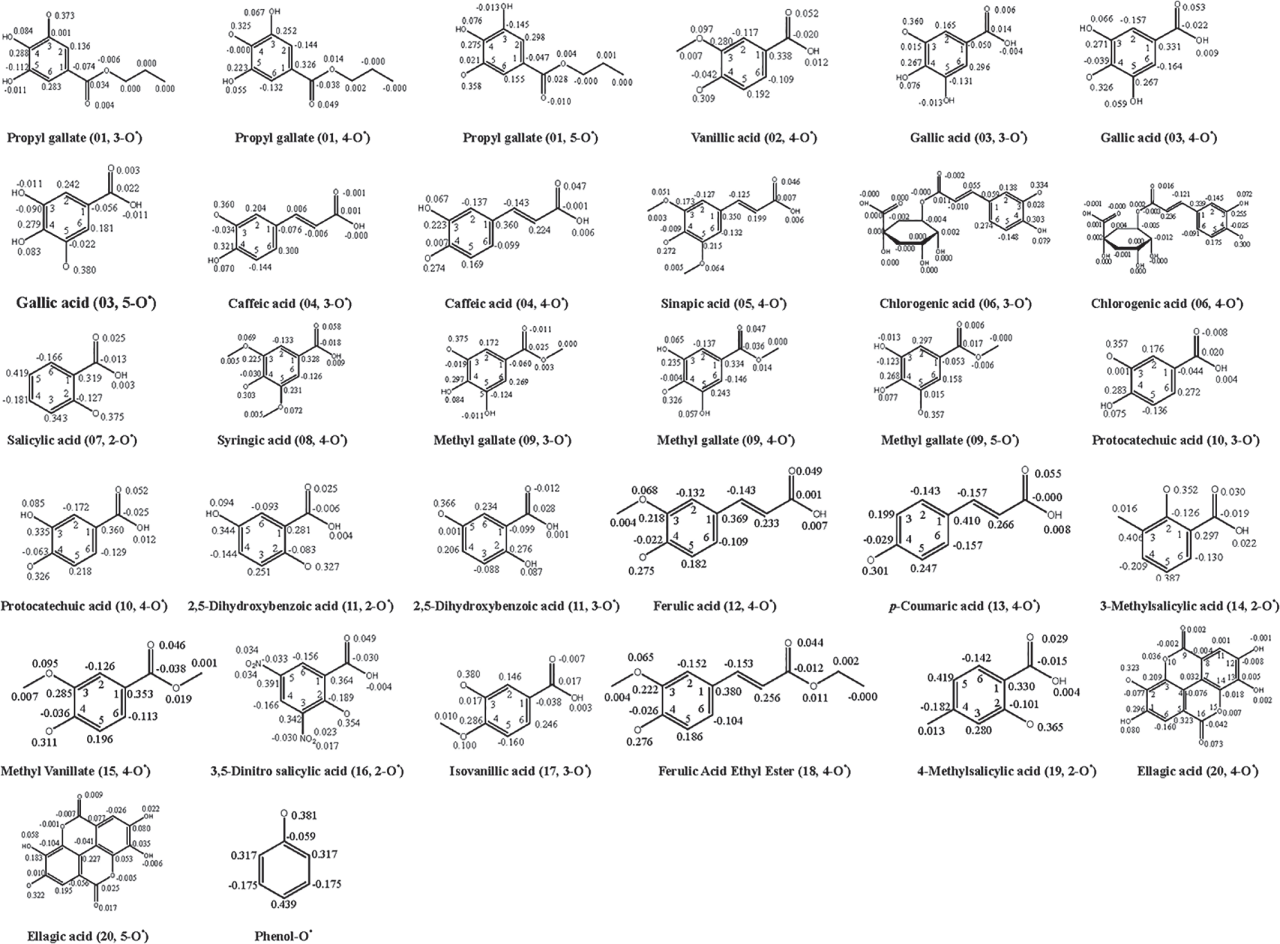
Spin density values of phenoxy radicals of 20 investigated phenolic compounds and phenol calculated at the B3LYP/6-311++G(d,p) levels of theory in ethanol.

We investigated the conformational, electronic, and geometrical features of phenolic systems, which are of crucial importance for understanding their structure-antioxidant activity relationships [10]. For three compounds containing 3 adjacent phenolic OHs, such as propyl gallate (01), gallic acid (03) and methyl gallate (09), the 4-OH BDE values are generally smaller than the corresponding 3-OH and 5-OH BDE values (Table 1). Moreover, the energies of the 4-O^•^ radicals are smaller than those of the 3-O^•^ and 5-O^•^ radicals (Table 2), leading to the fact that for the same compound, the O^•^ centers in the *para* position to 3 groups (-COOCH_2_CH_2_CH_3_, -COOH and COOCH_3_) are more stable. For the spin density distribution of propyl gallate (01) displayed in Fig. 3, the spin density (0.325) in the 4-O^•^ center is lower than those of the 3-O^•^ (0.373) and 5-O^•^ (0.358) centers. Similarly, gallic acid (03) has a lower spin density (0.326) in the 4-O^•^ center than those in the 3-O^•^ and 5-O^•^ centers (0.360 and 0.380), while methyl gallate (09) has a lower spin density of 0.326 in the 4-O^•^ center than those in the 3-O^•^ and 5-O^•^ centers (0.375 and 0.357). As can be seen, in the same molecule, a low spin density of the O^•^ center indicates that the spin density experiences a more extended electronic delocalization, which is conducive to the stability of the radicals, thus leading to the smaller the corresponding BDE value.

**Table 2:** Absolute (*E* in kcal/mol) and relative energies (Δ*E* in kcal/mol) of various hydrogen atom-abstracted radicals in 4 reaction environments calculated at the B3LYP/6-311++G(d, p) levels of theory^a^.

No.	Compound	*Gas-phase*		Benzene		Water		Ethanol	
		*E*	Δ*E*	*E*	Δ*E*	*E*	Δ*E*	*E*	Δ*E*
1	Propyl gallate								
	3-OH	-479295.6	5.8	-479289.0	6.8	-479295.6	5.8	-479295.0	5.9
	4-OH	-479301.4	0.0	-479295.8	0.0	-479301.4	0.0	-479300.9	0.0
	5-OH	-479298.6	2.8	-479294.2	1.6	-479298.6	2.8	-479298.2	2.7
2	Vanillic acid								
	4-OH	-382760.8	0.0	-382755.3	0.0	-382760.8	0.0	-382760.3	0.0
3	Gallic acid								
	3-OH	-405332.1	2.4	-405327.5	0.8	-405332.1	2.4	-405331.7	2.2
	4-OH	-405334.4	0.0	-405328.3	0.0	-405334.4	0.0	-405333.9	0.0
	5-OH	-405328.7	5.8	-405321.3	7.0	-405328.7	5.8	-405328.1	5.9
4	Caffeic acid								
	3-OH	-406675.3	6.8	-406667.4	9.8	-406675.3	6.8	-406674.6	7.0
	4-OH	-406682.1	0.0	-406677.2	0.0	-406682.1	0.0	-406681.7	0.0
5	Sinapic acid								
	4-OH	-503190.9	0.0	-503182.8	0.0	-503190.9	0.0	-503190.2	0.0
6	Chlorogenic acid								
	3-OH	-813856.7	5.4	-813845.6	5.8	-813856.7	5.4	-813855.7	5.5
	4-OH	-813862.2	0.0	-813851.4	0.0	-813862.2	0.0	-813861.2	0.0
7	Salicylic acid								
	2-OH	-310890.1	0.0	-310867.6	0.0	-310890.1	0.0	-310889.4	0.0
8	Syringic acid								
	4-OH	-454626.3	0.0	-454619.5	0.0	-454626.3	0.0	-454625.8	0.0
9	Methyl gallate								
	3-OH	-429977.0	5.8	-429944.3	32.7	-429977.0	5.8	-429976.5	5.9
	4-OH	-429982.8	0.0	-429977.0	0.0	-429982.8	0.0	-429982.3	0.0
	5-OH	-429980.4	2.4	-429976.2	0.8	-429980.4	2.4	-429980.0	2.3
10	Protocatechuic acid								
	3-OH	-358112.8	4.2	-358106.6	6.3	-358112.8	4.2	-358112.4	4.4
	4-OH	-358117.1	0.0	-358112.9	0.0	-358117.1	0.0	-358116.7	0.0
11	2,5-Dihydroxybenzoic acid								
	2-OH	-358111.3	0.0	-358104.7	0.0	-358111.3	0.0	-358110.8	0.0
	5-OH	-358110.3	1.0	-358103.5	1.2	-358110.3	1.0	-358109.8	1.0
12	Ferulic acid								
	4-OH	-431325.8	0.0	-431319.0	0.0	-431325.8	0.0	-431325.2	0.0
13	*p*-Coumaric acid								
	4-OH	-359459.5	0.0	-359454.2	0.0	-359459.5	0.0	-359459.0	0.0
14	3-Methylsalicylic acid								
	2-OH	-335552.0	0.0	-335547.1	0.0	-335552.0	0.0	-335551.6	0.0
15	Methyl Vanillate								
	4-OH	-407409.1	0.0	-407404.0	0.0	-407409.1	0.0	-407408.7	0.0
16	3,5-Dinitro salicylic acid								
	2-OH	-567602.1	0.0	-567594.3	0.0	-567602.1	0.0	-567601.4	0.0
17	Isovanillic acid								
	3-OH	-382758.3	0.0	-382754.3	0.0	-382758.3	0.0	-382758.0	0.0
18	Ferulic Acid Ethyl Ester								
	4-OH	-480634.7	0.0	-480628.3	0.0	-480634.7	0.0	-480634.2	0.0
19	4-Methylsalicylic acid								
	2-OH	-335551.1	0.0	-335546.0	0.0	-335551.1	0.0	-335550.7	0.0
20	Ellagic acid								
	4-OH	-714377.0	4.1	-714365.5	6.2	-714377.0	4.1	-714376.0	4.3
	5-OH	-714381.1	0.0	-714371.7	0.0	-714381.1	0.0	-714380.3	0.0
21	Phenol	-192533.3	0.0	-192530.3	0.0	-192533.3	0.0	-192533.0	0.0

^a^ relative to phenol.

The distinct extended delocalization affects the spin density distribution of the corresponding radicals, thereby to bring out different BDEs and subsequent radical-scavenging activities for these investigated phenolic compounds. The extended delocalization in these radical systems is composed by the radical center, whole aromatic ring, relevant phenolic hydroxyl(s), and (C = C)COOH and COOR group(s). We found that the C = O spin density in 4-O^•^ distinctly differed from that of 3-O^•^ and 5-O^•^. For example, propyl gallate (01) has a C = O spin density of (-0.038, 0.049) for 4-O^•^, (0.034, 0.004) for 3-O^•^, and (0.028, -0.010) for 5-O^•^, respectively. Similarly, the C = O spin densities of 4-O^•^, 3-O^•^ and 5-O^•^ are (-0.022, 0.053), (0.014, 0.006) and (0.022, 0.003) in Gallic acid (03) while (-0.036, 0.047), (0.025, -0.011) and (0.017, 0.006) in methyl gallate (09), respectively. Overall, the C = O in the carboxylic acid could greatly strengthen the *para* 4-O^•^ center to form a more electronic delocalization relative to the *meta* 3-O^•^ and 5-O^•^ center. The distinctly extended C = O delocalization can be seen in the 4-O^•^ centers of other 10 compounds (Fig. 3), including vanillic acid (02, 4-O^•^), caffeic acid (04, 4-O^•^), sinapic acid (05, 4-O^•^), chlorogenic acid (06, 4-O^•^), syringic acid (08, 4-O^•^), protocatechuic acid (10, 4-O^•^), ferulic acid (12, 4-O^•^), *p*-coumaric acid (13, 4-O^•^), methyl vanillate (15, 4-O^•^) and ferulic acid ethyl ester (18, 4-O^•^).

The CH = CH bridge in C = CCOOH or C = CCOOR takes part in the extended delocalization, particularly in its *para* O^•^ center. Relative to gallic acid (02), ferulic acid (12) possesses a conjugated CH = CH bridge between the phenyl and carbonyl group, which favors a resonance and conjugation effect on its *para* radical center. Therefore, the corresponding radical center has a lower spin density, leading to a lower 4-OH BDE value. Similarly, 4-O^•^ of caffeic acid (04) has a lower spin density (0.274) than 4-O^•^ (0.326) of protocatechuic acid (10), thus the OH BDE value (75.1 kcal/mol) of the former is the lower that of the latter (83.2 kcal/mol). The spin density distribution where the CH = CH bridge participates can be seen from other 4 radicals, including sinapic acid (05, 4-O^•^), chlorogenic acid (06, 4-O^•^), *p*-coumaric acid (13, 4-O^•^) and ferulic acid ethyl ester (18, 4-O^•^) as displayed in Fig. 3.

Orthodiphenolic functionalities also affected the structure-antioxidant activity relationships of 7 phenolic compounds, including propyl gallate (01), gallic acid (03), caffeic acid (04), chlorogenic acid (06), methyl gallate (09), protocatechuic acid (10) and ellagic acid (20). Orthodiphenolic functionalities could well secure a relatively complete spin density delocalization [13], which makes these compounds have higher antioxidant activities than the compounds with a single free hydroxy group on the ring. One of the most representative examples is that two orthodiphenolic functionalities of ellagic acid (20) mutually promote the extended spin density delocalization as shown in Fig. 3. This leads the 5-O^•^ radical of this compound to be more stable, and the compound has the highest antioxidant activity (pIC_50_ = 5.00) among all the investigated compounds.

For 4 compounds with low antioxidant activities, including salicylic acid (07, pIC_50_ = 1.10), 3-methylsalicylic acid (14, pIC_50_ = 1.15), 3,5-dinitro salicylic acid (16, pIC_50_ = 0.61), and 4-methylsalicylic acid (19, pIC_50_ = 0.75), one common feature is that each compound has a single hydroxyl group in the *ortho* position next to the COOH group on the phenolic ring. These four compounds have a lower hydrogen donating ability than phenol under 4 different reaction environments due to their higher BDE values than phenol. The spin density (0.381) of phenol-O^•^ is relatively close to those of 2-O^•^ of these 4 parent compounds (0.375, 0.352, 0.354 and 0.365) (Fig. 3). Although there is a certain delocalization involving the O^•^ center, the C atoms of the ring, and the C = O constitution in COOH, this delocalization is not significant.

### Structure-IP-PDE-activity relationships

Unlike BDEs, IPs appeared to be easily influenced by the solvent polarity since polar solvents may affect charge separation in a molecule [14]. As can be seen in Table 3, the IP values are sorted in the order of gas>benzene>ethanol>water for the same molecule, indicating the electron donating ability is more favorable in polar media. Cation radicals are sensitive to the polarity of different solvents, thus as expected, the IP values in water are lower than those in ethanol. As stated above, the IP is reaction enthalpies related to the first step of the SETPT mechanism, thus BDEs and IPs determine the thermodynamically preferred reaction pathway. The calculated IP values (Table 3) are significantly larger than the corresponding BDE values (Table 1), from the thermodynamic point of view, suggesting the occurrence probability of the HAT mechanism is greater than that of the SETPT mechanism in 4 reaction micro-environments. The PDEs under different media reveal different values, especially, significant average fall is found in water and ethanol. We also observed the PDEs in water are greater than those in ethanol, thus the proton dissociation ability of the studied compounds in ethanol is slightly stronger than that in water.

**Table 3:** IPs and PDEs of 20 investigated phenolic compounds and phenol calculated at the B3LYP/6-311++G(d,p) level ^a^.

No.	Compound	IP (kcal/mol)				PDE (kcal/mol)				pIC_50_
		Gas	Benzene	Ethanol	Water	Gas	Benzene	Ethanol	Water	
1	Propyl gallate	**184.7**	**159.1**	**125.6**	**117.5**					4.81
	3-OH					215.4	45.3	1.6	8.1	
	4-OH					**207.8**	**38.5**	-**4.3**	**2.3**	
	5-OH					208.6	40.1	-1.6	5.1	
2	Vanillic acid	**185.9**	**159.1**	**125.1**	**116.9**					2.44
	4-OH					**214.5**	**45.3**	**2.7**	**9.2**	
3	Gallic acid	**189.7**	**162.1**	**127.3**	**119.1**					4.70
	3-OH					203.4	38.9	-3.1	3.8	
	4-OH					**203.6**	**38.1**	-**5.3**	**1.4**	
	5-OH					211.5	45.1	0.6	7.2	
4	Caffeic acid	**182.5**	**156.0**	**121.8**	**113.6**					4.52
	3-OH					218.1	50.5	5.4	12.0	
	4-OH					**206.4**	**40.7**	-**1.6**	**5.2**	
5	Sinapic acid	**170.5**	**146.8**	**115.2**	**107.2**					4.19
	4-OH					**222.7**	**49.9**	**4.8**	**11.2**	
6	Chlorogenic acid	**180.1**	**157.6**	**122.5**	**114.3**					4.37
	3-OH					219.5	44.7	5.9	16.0	
	4-OH					**208.2**	**35.5**	**1.0**	**10.5**	
7	Salicylic acid	**196.9**	**168.4**	**133.2**	**125.0**					1.10
	2-OH					**210.7**	**61.0**	**1.5**	**7.9**	
8	Syringic acid	**177.3**	**152.8**	**121.0**	**113.1**					4.32
	4-OH					**218.7**	**45.8**	**2.0**	**8.3**	
9	Methyl gallate	**186.4**	**159.8**	**125.9**	**117.7**					4.74
	3-OH					214.3	71.4	1.6	8.1	
	4-OH					**206.4**	**38.7**	-**4.3**	**2.4**	
	5-OH					206.2	39.6	-2.0	4.8	
10	Protocatechuic acid	**190.9**	**162.7**	**127.6**	**119.4**					4.25
	3-OH					209.8	43.6	0.1	6.7	
	4-OH					**201.1**	**37.2**	-**4.0**	**2.9**	
11	2,5-Dihydroxybenzoic acid	**184.6**	**156.5**	**121.7**	**113.5**					4.53
	2-OH					**210.6**	**46.0**	**2.1**	**8.8**	
	5-OH					237.3	47.2	3.1	9.7	
12	Ferulic acid	**177.7**	**152.3**	**119.2**	**111.1**					3.57
	4-OH					**219.6**	**49.1**	**5.2**	**11.7**	
13	*p*-Coumaric acid	**185.1**	**158.3**	**124.3**	**116.2**					4.54
	4-OH					**212.1**	**45.0**	**2.4**	**9.1**	
14	3-Methylsalicylic acid	**190.6**	**163.3**	**128.9**	**120.7**					1.15
	2-OH					**214.8**	**45.9**	**3.8**	**10.4**	
15	Methyl Vanillate	**182.6**	**156.9**	**123.7**	**115.6**					4.60
	4-OH					**217.3**	**45.7**	**3.5**	**10.0**	
16	3,5-Dinitro salicylic acid	**232.6**	**202.7**	**163.1**	**154.4**					0.61
	2-OH					**180.3**	**24.7**	-**20.9**	-**13.7**	
17	Isovanillic acid	**184.3**	**157.8**	**124.3**	**116.2**					2.33
	3-OH					**209.6**	**41.6**	**0.2**	**6.9**	
18	Ferulic Acid Ethyl Ester	**174.3**	**150.3**	**117.9**	**109.9**					3.61
	4-OH					**222.5**	**49.4**	**6.0**	**12.4**	
19	4-Methylsalicylic acid	**193.5**	**166.2**	**131.6**	**123.5**					0.75
	2-OH					**206.7**	**40.3**	-**2.1**	**4.6**	
20	Ellagic acid	**182.1**	**157.7**	**124.6**	**116.4**					5.00
	4-OH					217.8	51.7	3.0	9.6	
	5-OH					**210.2**	**45.5**	-**1.3**	**5.5**	
21	Phenol	**192.9**	**163.2**	**127.7**	**119.5**	205.9	17.6	0.2	7.0	

^a^ The parameters are calculated based on the optimized structures with intra-molecular hydrogen bonds. The bold parameters are used as independent variables in the QSAR model.

The explored compounds have different reaction capability for the SETPT mechanism due to their different IP values (Table 3) in 4 different environments. It should be noted that 4 compounds, including salicylic acid (07), 3-methylsalicylic acid (14), 3,5-dinitro salicylic acid (16) and 4-methylsalicylic acid (19), have larger IPs than phenol. Together with the evidence of the 4 compounds with larger BDE values than phenol (Table 1), we concluded the 4 compounds, which all possess a single phenolic OH group in the *ortho* position to the COOH group, have a similar reactivity tendency for both the HAT and SETPT mechanisms. In particular, 3,5-dinitro salicylic acid (16) has both the largest BDE values (97.1 and 97.2 kcal/mol, Table 1) and the largest IP values (154.4 and 163.1 kcal/mol, Table 3) in water and ethanol, respectively, demonstrating this compound has a lowest reaction probability for both the HAT and SETPT mechanisms in polar solution. Two strong electron-drawing NO_2_ groups in this compound may induce a reduction in the electron density of the phenolic ring, so that this compound has a lowest reaction probability of the two mechanisms among all the investigated compounds.

### Structure-PA-ETE-activity relationships

According to the SPLET mechanism, we examined the deprotonation of phenolic OH group(s) and electron transfer tendency in the gas phase and solvents by calculating PAs and ETEs. Table 4 displays the PA values decrease significantly in the order of gas>benzene>water>ethanol for the same molecule, due to the high solvation enthalpies of proton. Therefore, the deprotonation process is more likely to occur in polar solvents, such as water and ethanol. A number of studies have shown that solvents induce significant changes in enthalpies of charged species, which dominantly affects SETPT and SPLET energetics [18,41]. In benzene, PAs (Table 4) are lower than IPs (Table 3), but still higher than the corresponding BDEs (Table 1). By combining with the analysis above for both the HAT and SETPT mechanisms, HAT appears to represent the most thermodynamically probable reaction pathway. However, in water and ethanol, PAs are considerably lower than BDEs and IPs, thus SPLET represents the thermodynamically preferred mechanism. In comparison to the IP values (Table 3) of the neutral form, the corresponding ETE values are significantly lower. Hence, the single electron transfer process from the anionic form is more preferable than that from the neutral form.

**Table 4:** PAs and ETEs of 20 investigated phenolic compounds and phenol calculated at the B3LYP/6-311++G(d,p) level ^a^.

No.	Compound	PA (kcal/mol)				ETE (kcal/mol)				pIC_50_
		Gas	Benzene	Ethanol	Water	Gas	Benzene	Ethanol	Water	
1	Propyl gallate									4.81
	3-OH	344.2	101.6	42.6	46.3	55.9	79.3	84.6	79.8	
	4-OH	**330.2**	**90.2**	**34.4**	**38.5**	**62.4**	**81.4**	**86.9**	**84.4**	
	5-OH	330.1	90.7	35.9	40.1	63.3	82.5	88.1	85.5	
2	Vanillic acid									2.44
	4-OH	**336.7**	**95.6**	**38.8**	**42.8**	**63.7**	**83.4**	**88.9**	**86.2**	
3	Gallic acid									4.70
	3-OH	327.7	88.8	35.1	39.4	65.4	83.5	89.1	87.3	
	4-OH	**328.0**	**88.2**	**33.2**	**37.4**	**65.3**	**83.1**	**88.8**	**87.1**	
	5-OH	342.7	100.4	41.8	45.6	58.4	80.7	86.1	81.9	
4	Caffeic acid									4.52
	3-OH	339.2	99.1	42.3	46.2	61.5	79.4	84.9	82.6	
	4-OH	**318.6**	**82.4**	**31.1**	**35.6**	**70.3**	**83.3**	**89.1**	**89.5**	
5	Sinapic acid									4.19
	4-OH	**332.0**	**93.5**	**38.2**	**42.3**	**61.2**	**76.1**	**81.8**	**80.9**	
6	Chlorogenic acid									4.37
	3-OH	325.2	80.3	32.8	39.7	69.4	82.0	88.9	89.7	
	4-OH	**319.8**	**78.9**	**29.4**	**33.9**	**78.5**	**90.2**	**92.6**	**92.9**	
7	Salicylic acid									1.10
	2-OH	**341.8**	**99.7**	**42.5**	**46.4**	**65.8**	**86.5**	**92.3**	**106.4**	
8	Syringic acid									4.32
	4-OH	**337.5**	**96.3**	**39.0**	**42.9**	**58.5**	**78.6**	**84.0**	**80.9**	
9	Methyl gallate									4.74
	3-OH	344.2	101.6	42.5	46.2	56.5	79.7	85.0	106.0	
	4-OH	**329.9**	**90.0**	**34.4**	**38.4**	**62.9**	**81.7**	**87.2**	**84.9**	
	5-OH	329.1	89.9	35.7	40.0	63.5	82.6	88.2	85.8	
10	Protocatechuic acid									4.25
	3-OH	343.2	100.8	42.5	46.3	57.5	81.1	85.2	79.8	
	4-OH	**323.3**	**84.7**	**31.6**	**35.9**	**68.6**	**90.5**	**92.1**	**86.4**	
11	2,5-Dihydroxybenzoic acid									4.53
	2-OH	**335.7**	**94.7**	**38.9**	**43.0**	**59.5**	**79.3**	**84.8**	**82.4**	
	5-OH	335.6	100.6	43.4	47.4	86.3	75.8	81.3	77.6	
12	Ferulic acid									3.57
	4-OH	**331.2**	**92.8**	**38.1**	**42.2**	**66.1**	**80.6**	**86.3**	**85.9**	
13	*p*-Coumaric acid									4.54
	4-OH	**326.5**	**89.1**	**36.2**	**40.5**	**70.6**	**84.8**	**90.5**	**90.4**	
14	3-Methylsalicylic acid									1.15
	2-OH	**341.6**	**100.2**	**43.3**	**47.2**	**63.9**	**83.9**	**89.4**	**86.5**	
15	Methyl Vanillate									4.60
	4-OH	**338.5**	**97.2**	**39.9**	**43.8**	**61.3**	**81.8**	**87.3**	**83.9**	
16	3,5-Dinitro salicylic acid									0.61
	2-OH	**295.9**	**62.2**	**14.3**	**19.1**	**116.9**	**121.6**	**127.9**	**132.9**	
17	Isovanillic acid									2.33
	3-OH	**335.6**	**95.4**	**39.6**	**43.7**	**58.3**	**79.4**	**84.9**	**81.2**	
18	Ferulic Acid Ethyl Ester									3.61
	4-OH	**333.0**	**94.4**	**39.1**	**43.1**	**63.8**	**79.2**	**84.8**	**83.7**	
19	4-Methylsalicylic acid									0.75
	2-OH	**335.3**	**94.4**	**38.2**	**42.3**	**64.9**	**85.8**	**91.3**	**88.0**	
20	Ellagic acid									5.00
	4-OH	322.7	85.3	31.4	35.6	77.2	90.4	96.2	96.2	
	5-OH	**314.5**	**79.8**	**29.3**	**33.8**	**77.8**	**88.1**	**94.0**	**95.6**	
21	Phenol	345.1	102.5	44.2	48.1	52.2	78.4	83.7	78.4	

^a^ The parameters are calculated based on the optimized structures with intra-molecular hydrogen bonds. The bold parameters are used as independent variables in the QSAR model.

All the compounds have lower PA values than phenol (345.1, 102.5, 48.1 and 44.2 kcal/mol) in 4 different environments (Table 4), revealing the compounds possess the ability to scavenge DPPH radicals to some degrees as they take effects on the radical scavenging by the SPLET mechanism, if possible. Three compounds, including salicylic acid (07, pIC_50_ = 1.10), 3-methylsalicylic acid (14, pIC_50_ = 1.15) and 4-methylsalicylic acid (19, pIC_50_ = 0.75), have relatively high PA values (42.5, 43.3 and 38.2 kcal/mol) in ethanol (Table 4), suggesting these compounds are not susceptible to the SPLET mechanism. As described above, the 3 compounds were unfavorable for both the HAT and SETPT mechanisms, because the 3 compounds with a common characteristic, i.e., a single OH group in the *ortho* position to the COOH group, have relatively low antioxidant activities in ethanol.

One of interesting findings is that 3,5-dinitro salicylic acid (16) has the lowest PA values (295.9, 62.2, 19.1 and 14.3 kcal/mol), suggesting this compound can release H^+^ from the 2-OH group much easier than other compounds. Two NO_2_ groups, as strong electron-withdrawing groups contained in this compound, bring out a reduction in the electron density of the phenol ring, thereby making the strongest H^+^ donation ability. The first step is easy to occur, however, the ETE value of the second step is the highest among all the compounds, meaning that the second step is very difficult to react, as evidenced by a relatively low activity (pIC_50_ = 0.75). In short, substituents with different electronegativities can alter the electron density of the phenolic ring, thereby to influence the abstraction of H^•^ and H^+^ release, and the occurrence probability of the individual mechanism.

### HOMO orbitals

**Fig 4:**
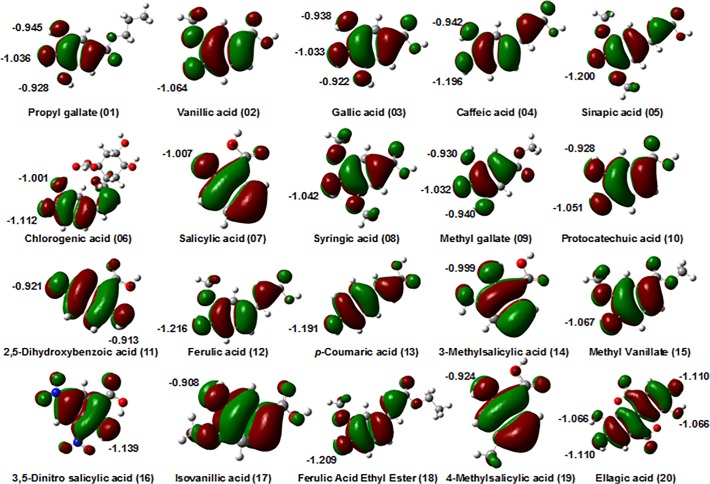
HOMO of 20 investigated phenolic compounds calculated at the B3LYP/6-311++G(d,p) levels of theory in ethanol. The numbers indicates atomic polar tensor charges.

Within the framework of molecular orbital theory, important information on the working mechanisms of antioxidants can be derived from the frontier highest occupied molecular orbital (HOMO) energy. Generally, the lower the HOMO energy is, the weaker the molecule donating electron ability is. On the contrary, a higher HOMO energy implies that the molecule is a good electron donor [42]. Because the H abstraction reaction involves electron transfer, the HOMO composition of a phenolic compound can provide a qualitative data to identify its active site for the scavenging radical activity [11,43]. For the compounds with two or more phenol OH groups, Fig. 4 shows there are more atomic polar tensors [44] atomic charges in the more probable electron-donating sites; moreover, those easily attacked sites consistently respond to the stable O^•^ centers with small spin density values as displayed in Fig. 3.

### Interpretation of action mechanisms by QSAR models

On the basis of the calculated thermodynamics parameters in polar media (water and ethanol) by DFT calculations, we established QSAR models to explore the characterization ability of the parameters on the reaction characteristics, thereby to further help interpret the mechanisms of the studied compounds. Table 5 displays all the QSAR models exhibit qualified fitting and predictive abilities because regression modeling coefficients and cross-validated correlation coefficients fall in the range of *Q* ^2^>0.500 and *R* ^2^>0.600 [45], respectively. These demonstrated five sets of calculated thermodynamics parameters could characterize the antioxidant activities of the compounds, and our QSAR models favorably describe the thermodynamics-activity relationships of these natural phenolic compounds in polar media. Particularly, the PA-ETE-based models have larger *R* ^2^_water_ of 0.739, *R* ^2^_ethanol_ of 0.745, *Q* ^2^_water_ of 0.674 and *Q* ^2^_ethanol_ of 0.689) than those of the BDE-based and IP-PDE-based models, suggesting PAs and ETEs are more effective descriptors for explaining the radical scavenging activities of these compounds relative to BDE and IP-PDE descriptors in polar solvents [11].

**Table 5:** QSAR modeling results of the thermodynamics-activity relationship for 20 phenolic compounds in water and ethanol ^a^.

Medium	Coefficient		*t*-test	*R* ^2^_rm_	*F*-test_rm_	*SD*_rm_	*Q* ^2^_cv_	*F*-test_cv_	*SD*_cv_
Water	Constant	21.765	/	0.691	40.072	0.886	0.588	25.686	0.998
	BDE	-0.227	-6.330						
Ethanol	Constant	21.584	/	0.689	39.928	0.867	0.594	26.291	0.991
	BDE	-0.224	-6.319						
Water	Constant	34.359	/	0.684	18.394	0.899	0.526	9.418	1.102
	IP	-0.246	-5.753						
	PDE	-0.292	-4.000						
Ethanol	Constant	34.973	/	0.711	20.940	0.860	0.577	11.611	1.040
	IP	-0.250	-6.185						
	PDE	-0.325	-4.391						
Water	Constant	34.721	/	0.739	23.021	0.821	0.674	17.001	0.998
	PA	-0.293	-5.473						
	ETE	-0.238	-6.897						
Ethanol	Constant	36.150	/	0.745	24.801	0.808	0.689	18.857	0.892
	PA	-0.299	-5.873						
	ETE	-0.244	-7.042						

^*a*^ *The R* ^2^_rm_, *F*-test_rm_, *SD*_rm_, *Q* ^2^_cv_, *F*-test_cv_ and *SD*_cv_ are the multiple correlation coefficient, Fisher’s criterion, and standard error by regression modeling and leave-one-out cross validation, respectively.

As shown in Table 5, the absolute values of the *t*-test values for all the variables (BDE, IP, PDE, PA and ETE) are larger than 2.000, thus the variables significantly influence the dependent variables (pIC_50_). Each variable in the QSAR models has a negative coefficient, indicating the variables have negative effects on the radical scavenging activities, consistent with the well known fact that the smaller the thermodynamic parameters, the larger the corresponding activities. We found the PDE coefficients (−0.292 and −0.325) are smaller than the IP coefficients (-0.246 and -0.250) for the IP-PDE-activity models in both water and ethanol, while the PA coefficients (-0.293 and -0.299) are smaller than the ETE coefficients (-0.238 and -0.244) for the PA-ETE-activity models in both water and ethanol. Therefore, the proton dissociation ability plays a significant effect on the activities of the explored phenolic compounds in polar media such as water and ethanol.

Our QSAR modeling results reveal the fitting and predictive abilities of the PA-ETE-activity models are distinctly higher those of BDE-activity models (Table 5), which is supported by the conclusion on the working mechanisms of the explored compounds by DFT calculations, specifically, from the thermodynamic point of view, the SPLET mechanism would play a dominant role in water and ethanol. Therefore, one could try to choose the PA-ETE-activity model to predict antioxidant activities of new phenolic acids and derivatives in polar solvents.

## Conclusions

We employ a combination of computational and experimental approaches, including DPPH^•^ scavenging activity determination, DFT calculations and QSAR modeling, to investigate the structure-thermodynamic-antioxidant activity relationships of 20 phenolic acids and derivatives in different reaction environments. The conclusions are as follows: (1) The phenolic radicals can be stabilized by two main factors: the possibility to establish intra-molecular hydrogen bonds and the extended delocalization and conjugation of the electrons enhanced by resonance phenomena. (2) These calculated descriptors (BDE, IP, PDE, PA and ETE) can be used to effectively describe the mechanisms (HAT, SETPT and SPLET) of the studied compounds in individual micro-environments, which demonstrates HAT is the more favorable mechanism in the gas phase and benzene, whereas the SPLET mechanism prefers in water and ethanol. (3) Our QSAR models can characterize the structure-antioxidant activity relationships of the studied compounds in polar media.

Our future work will focus on the structure-activity relationships of more phenolic acids and derivatives using combined experimental and computational approaches. Investigations of the reaction kinetics of the phenolic compounds with DPPH^•^ are of significance not only for enriching the knowledge of chemical mechanism(s) of action, but also for stimulating the discovery of effective drugs, food additives, or other functional molecules.

## Supporting Information

S1 File Table A. Structures and experimental radical scavenging activities of selected phenolic acids and derivatives. **Fig. A**. Optimized structures of seven compounds with hydrogen bond(s) between orthodiphenolic functionalities calculated at the B3LYP/6-311++G(d,p) levels of theory in ethanol.
(DOC)
