# Supplementary material for: Structure-Thermodynamics-Antioxidant Activity Relationships of Selected Natural Phenolic Acids and Derivatives: An Experimental and Theoretical Evaluation
Source: PLoS One. 2015 Mar 24;10(3):e0121276. doi: 10.1371/journal.pone.0121276 (PMC4372407; doi:10.1371/journal.pone.0121276)
Supplement: S1 File — Structures and experimental radical scavenging activities of selected phenolic acids and derivatives. Fig. A. Optimized structures of seven compounds with hydrogen bond(s) between orthodiphenolic functionalities calculated at the B3LYP/6-311++G(d,p) levels of theory in ethanol. (DOC) [file pone.0121276.s001.doc]

***Supporting Information***

**Structure-thermodynamics-****antioxidant Activity Relationships of Selected Natural Phenolic Acids and Derivatives: An Experimental and Theoretical Evaluation**

**Yuzhen Chen^1^, Huizhi Xiao^2^, Jie Zheng^3^ and Guizhao Liang^2*^**

**1** School of Mathematical Sciences, Henan Institute of Science and Technology, Xinxiang 453003, P. R. China

**2** Key Laboratory of Biorheological Science and Technology, Ministry of Education, School of Bioengineering, Chongqing University, Chongqing 400044, P. R. China

**3** Department of Chemical and Biomolecular Engineering, The University of Akron, Akron, Ohio 44325, United States

* E-mail: [gzliang@cqu.edu.cn](mailto:gzliang@cqu.edu.cn).

YZC and HZX contributed equally to this work.

**Table A. Structures and experimental radical scavenging activities of selected phenolic acids and derivatives**

| No. | Compound | IC_50_(μg/mL) | pIC_50_ |
| --- | --- | --- | --- |
| 1 | Propyl gallate | 3.31 | 4.81 |
| 2 | Vanillic acid | 606.45 | 2.44 |
| 3 | Gallic acid | 3.43 | 4.70 |
| 4 | Caffeic acid | 5.48 | 4.52 |
| 5 | Sinapic acid | 14.54 | 4.19 |
| 6 | Chlorogenic acid | 15.25 | 4.37 |
| 7 | Salicylic acid | 10921.06 | 1.10 |
| 8 | Syringic acid | 9.38 | 4.33 |
| 9 | Methyl gallate | 3.32 | 4.74 |
| 10 | Protocatechuic acid | 8.57 | 4.26 |
| 11 | 2,5-Dihydroxybenzoic acid | 4.54 | 4.53 |
| 12 | Ferulic acid | 52.79 | 3.57 |
| 13 | *p*-Coumaric acid | 4.72 | 4.54 |
| 14 | 3-Methylsalicylic acid | 10824.58 | 1.15 |
| 15 | Methyl Vanillate | 4.58 | 4.60 |
| 16 | 3,5-Dinitro salicylic acid | 55403.16 | 0.62 |
| 17 | Isovanillic acid | 788.5 | 2.33 |
| 18 | Ferulic Acid Ethyl Ester | 54.56 | 3.61 |
| 19 | 4-Methylsalicylic acid | 27175.43 | 0.75 |
| 20 | Ellagic acid | 3.02 | 5.00 |

**Figure A. Optimized structures of seven compounds with hydrogen bond(s) between orthodiphenolic functionalities calculated at the B3LYP/6-311++G(d,p) levels of theory in ethanol.**
